# Past and Future Influenza Vaccine Uptake Motivation: A Cross-Sectional Analysis among Italian Health Sciences Students

**DOI:** 10.3390/vaccines11040717

**Published:** 2023-03-23

**Authors:** Pasquale Stefanizzi, Sandro Provenzano, Omar Enzo Santangelo, Giulia Dallagiacoma, Vincenza Gianfredi

**Affiliations:** 1 Interdisciplinary Department of Medicine, University of Bari Aldo Moro, Piazza G. Cesare 11, 70121 Bari, Italy; 2 Local Health Unit of Trapani, ASP Trapani, 91100 Trapani, Italy; 3 Regional Health Care and Social Agency of Lodi, ASST Lodi, Piazza Ospitale 10, 26900 Lodi, Italy; 4 Department of Public Health, Experimental and Forensic Medicine, School of Public Health, University of Pavia, 27100 Pavia, Italy; 5 Department of Global Public Health, Karolinska Institute, 171 77 Stockholm, Sweden; 6 Department of Biomedical Sciences for Health, University of Milan, Via Pascal 36, 20133 Milan, Italy

**Keywords:** influenza vaccines, vaccination hesitancy, medical students, information sources

## Abstract

Despite its effectiveness in the prevention of seasonal flu, influenza vaccination uptake remains low, even among healthcare workers (HCWs), despite their occupational risk. The aim of this study was to explore the association between main reasons for accepting or refusing influenza vaccination and the decision to receive the vaccination during both previous and following year among health sciences students. A multi-center, cross-sectional study was performed using a validated online questionnaire. Data were analyzed by performing univariable and multivariable logistic analysis. Data from over 3000 participants showed that avoiding the spread of infection to family members and the general population (aOR: 43.55), as well as to patients (aOR: 16.56) were the main reasons associated with the highest probability of taking the influenza vaccination the following year. On the contrary, not considering influenza as a severe disease was the reason associated with the lowest probability for past (aOR: 0.17) and future vaccination (aOR: 0.01). Therefore, the importance of vaccination to protect others should always be the core of vaccination campaigns for health sciences students, together with tools to increase their awareness of the severity of this disease.

## 1. Introduction

The human right to health has been recognized by the World Health Organization (WHO) as an individual right, and its protection and promotion involve not only institutions but also the whole community. Vaccinations are a key component of primary healthcare and are universally recognized as one of the most effective preventive measures in public health [1]. Along with sanitation and access to clean drinking water, vaccination represents one of the most successful public health interventions that are undeniably responsible for improved health outcomes globally [2]. Yet, despite the tremendous scientific advances, global immunization coverage has plateaued over the last decades [3]. This was further worsened over the last few years by the prolonged suspension of immunization services due to the overwhelming pressure on health systems globally during the COVID-19 pandemic [4,5]. Vaccine-preventable diseases (VPDs), including influenza, therefore represent a significant source of both morbidity and mortality in the general population, especially among those suffering from high-risk medical conditions and chronic diseases (e.g., cancer patients) [6,7]. In this context, the Global Vaccine Action Plan—endorsed by the 194 Member States of the World Health Assembly in May 2012—is a framework developed with the purpose of creating a world free of VPDs through more equitable access to existing vaccines for people in all communities [8]. In fact, both globally and at the European level, the importance of implementing immunization policies and targeted strategies needs to be addressed to counteract the decline in vaccine coverage rate and the risk of VPD outbreaks, which would put additional pressure on healthcare systems [9]. In the last decades, a general decline in coverage rate has been observed in several European countries and for multiple VPDs, including influenza and measles [10], causing new outbreaks such as the Italian measles epidemic in 2017 [11]. Influenza is a highly contagious yet preventable acute respiratory illness that affects approximately 5–10% of the general population every year. In industrialized countries, influenza vaccines offer approximately 70–90% protection against clinical disease in healthy adults. Influenza vaccines are considered safe, as serious adverse events are rarely reported, and vaccination against influenza represents the most effective way to reduce morbidity and mortality, especially among high-risk groups [12,13,14,15]. Among non-institutionalized elderly people, vaccination may reduce the number of hospitalizations by 25–39% and the overall mortality by 39–75% during influenza seasons [16]. The decline in vaccine coverage has highlighted the need for national immunization programs to develop approaches and strategies to address the increasing issue of vaccine hesitancy [17]. Safe and effective vaccines to prevent seasonal influenza infection are available, yet a significant proportion of the population remains unvaccinated, and many have no intention of receiving the vaccine. In Italy, the Ministry of Health publishes official recommendations through the National Vaccine Prevention Plan (Piano Nazionale Prevenzione Vaccinale, PNPV), the latest being PNPV 2023–2025, to maintain optimal vaccination coverage rates and comply with regional strategies. The PNPV is a guidance document on immunization policies, which includes a list of all vaccines (including influenza vaccination) that are provided free of charge to specific groups of people who are at higher risk due to factors such as age, professional exposure or health conditions [18]. Moreover, the PNPV was designed to identify professionals who may be exposed to biological agents or have a high risk of contracting infectious diseases due to their occupation. Health care workers (HCWs) are among the at-risk professionals included in this group. In fact, the Strategic Advisory Group of Experts on Immunization (SAGE) of the WHO recommends prioritizing influenza vaccination for HCWs, as well as for adults aged 65 and above, individuals with underlying health conditions, pregnant women and children aged 6–59 months [19]. According to the Centers for Disease Control and Prevention (CDC), HCWs include a diverse range of professionals such as physicians, nurses, emergency medical personnel, dental professionals and students, medical and nursing students, laboratory technicians, pharmacists, hospital volunteers, and administrative staff: all of these are considered a priority target group for influenza vaccination [20]. It is widely acknowledged that HCWs should receive one dose of influenza vaccine annually due to their occupational exposure, both to protect themselves and because they may act as vectors in the nosocomial transmission of influenza, especially for immunocompromised patients, but also for co-workers, friends and relatives [21,22]. Although achieving high vaccination coverage in target population groups can significantly reduce morbidity, complications and mortality related to influenza, the percentage of vaccinated individuals worldwide remains low [21]. According to the Italian Ministry of Health, the minimum vaccination coverage rate for the target population is 75%, while the optimal rate is 95% to achieve effective protection against influenza. Nevertheless, during 2021/2022 influenza season, the vaccination coverage in Italy was around 20.5% among the general population, and 58.1% in people older than 65 years, which is a decrease of 7 percentage points compared to the previous season [23]. There is currently no available data in Italy about the coverage of seasonal influenza vaccination among HCWs. However, studies have shown that knowledge and coverage of recommended vaccinations in this high-risk group are often insufficient [24,25]. A recent Italian study conducted among HCWs of the IRCCS Ospedale Policlinico San Martino in Genoa revealed that influenza vaccinations increased from 12.8% in 2019/2020 to 40.9% during the 2020/2021 influenza season (*p* < 0.01). However, the rate subsequently fell to 23.0% in the 2021/2022 influenza season (*p* < 0.01) [26]. These values are far below the minimum rate of influenza vaccination coverage reported by the Ministry of Health [23].

The aim of the current survey was to investigate the relationship between the primary reasons for accepting or refusing influenza vaccination and the decision to receive the vaccine during both the previous and the following year, among Italian health professions students.

## 2. Materials and Methods

### 2.1. Study Design

This is a multi-center (14 Italian Universities: Ancona, Bari, Naples, L’Aquila, Messina, Palermo, Parma, Pavia, Perugia, Rome, Salerno, Siena, Turin, Udine), cross-sectional study based on an electronic survey developed by the “Vaccination and vaccine hesitancy” working group of the Committee of Medical Residents of the Italian Society of Hygiene and Preventive Medicine. Full methodological details are available in a previously published paper [27]. The validated questionnaire was administered to university students of health sciences degree programs, that are provided by the faculty of Medicine, including: medicine and surgery, dentistry, nursing, physiotherapy, exercise science, pharmacy, environment and workplace prevention techniques. More details are reported in Supplementary Table S1. The questionnaire was a multiple-choice items (close-ended), including the following sections: (i) demographic data: sex, age, degree program and year, university; (ii) self-reported level of knowledge on vaccines and vaccine preventable diseases, (iii) personal experiences of influenza vaccination; (iv) reasons for getting or not getting vaccinated against influenza, (v) main sources of information on influenza vaccination; (vi) attitudes to recommend influenza vaccination; and (vii) willingness to participate in a specific course on influenza and influenza vaccination [28]. In the questionnaire, the term “previous year” refers to the previous influenza vaccination campaign, while the “following year” refers to the following influenza vaccination campaign. All data are self-reported. The recruitment was carried out by members of the study team during a lecture on preventive medicine. During the lecture, the study’s aim was presented and the students were invited to complete the questionnaire. They were shown a quick response code that redirected them to the online version of the questionnaire. Each member was in charge of recruiting participants from their own university. The recruitment took place from October 2017 to September 2018.

### 2.2. Ethical Considerations

Informed consent was obtained from all participants. The validated, 21-item online questionnaire was created using Google Forms^®^ and was accessible through a Quick Response (QR) code. All data collected were stored in an electronic database protected by a password known only to the data manager. Ethical approval was given by the local Ethical Committee of the University of Perugia (Comitato Universitario di Bioetica), Reference Number 2017-20R. Ethical approval was subsequently provided from each local Ethical Committee of the included universities.

### 2.3. Statistical Analysis

For all qualitative variables, absolute and relative frequencies were calculated; categorical variables were analyzed by Pearson’s Chi-square test (χ2). Student’s *t*-test and standard deviation were calculated for the means. The degree course variable has been dichotomized into “Medicine and Surgery” and “others” (for more detail about “others”, see Supplementary Table S1). A multivariable logistic regression model was used, and the chosen dependent variables were “having being vaccinated against influenza the previous year” and “planning on receiving the influenza vaccination the following year”. For selected dependent variables, adjusted odds ratios (aOR) are also presented. Each independent variable in the model was adjusted for age and gender. Results are expressed as adjusted Odds Ratio (aOR) with 95% Confidence Intervals (95% CI). The level of significance chosen for statistical analysis was 0.05. Analyses were conducted using statistical software STATA^®^ version 14 (StataCorp LLC, College Station, TX, USA).

## 3. Results

### 3.1. Descriptive Characteristics

A total of 3137 questionnaires were obtained; however, six of them were incorrectly filled out and therefore excluded from the analysis. Table 1 shows the general characteristics of the study population, stratified for influenza vaccination status during the previous year and intention to receive it during the following campaign. Participants had a mean age of 23.41 ± 3.69 years and 68.09% were female. A total of 349 (11.15%) students had received the influenza vaccination the previous year, 1093 (34.91%) students intended to receive the influenza vaccination during the following year, and a total of 337 (96.56%) students were both vaccinated the previous year and intended to get vaccinated during the following year. Participants who had received the influenza vaccination during the previous year were more frequently medical students and used institutional webpages and scientific data as main sources of vaccine-related information. Similar data were obtained among those who were planning on getting vaccinated during the following campaign.

**Figure 1:**
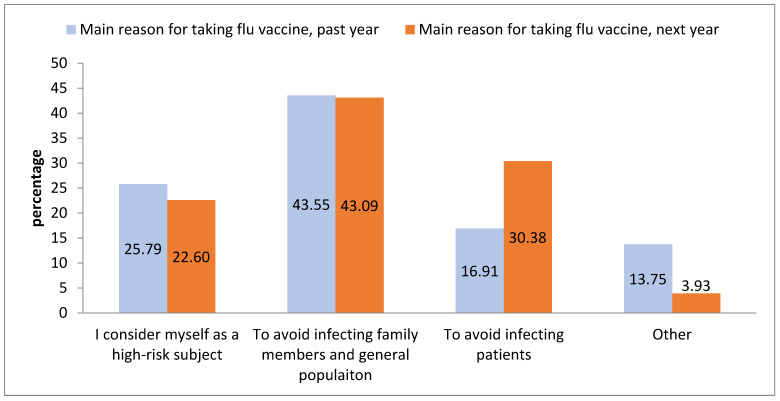
Comparing main reason for taking the vaccine the previous/following year. Used Pearson’s chi-squared test, chi-squared *p*-value < 0.001.

Figure 1 shows the distribution of the main reasons for taking the vaccination, both the previous year and for the following year. Among those who received the vaccination the previous year, the most frequently reported reason was to avoid spreading the infection to family members and to the general population (43.55%), followed by considering themselves high-risk subjects (25.79%). Considering those who were planning on receiving the vaccination during the following campaign, avoiding spreading the infection to family members and the general population (43.09%), and avoiding spreading the infection to patients (30.38%) were the two most frequently reported reasons.

**Figure 2:**
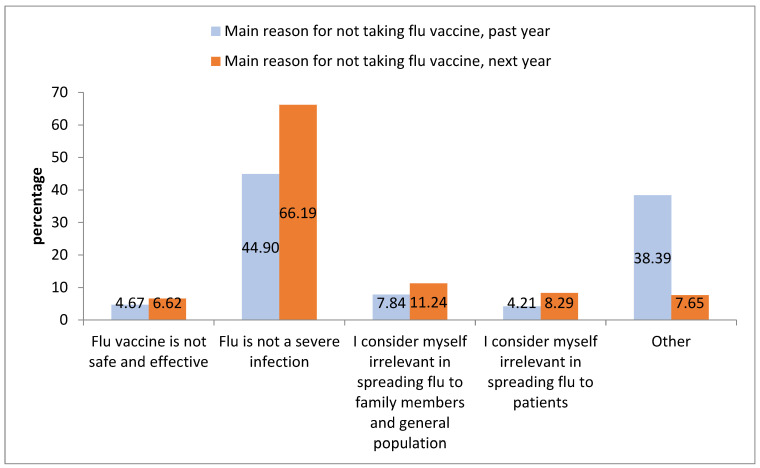
Comparing main reason for not taking influenza vaccine the previous/following year. Used Pearson’s chi-squared test, chi-squared *p*-value < 0.001.

Figure 2 shows the distribution of the main reasons for not taking the vaccination both the previous and the following year. In both cases, the fact that influenza was not considered as a severe infection was the main reason for refusing vaccination (44.90% for the previous year; 66.19% for the following year).

### 3.2. Influenza Vaccine Acceptance during the Previous Year

For each unit increase in age, the probability of accepting the influenza vaccine during the past year increased in a statistically significantly manner (aOR: 1.02; 95% CI: 1.01–1.04; *p* = 0.004). Comparing students enrolled in a medical degree course with all the others showed that those enrolled in other health sciences programs were less likely to have been vaccinated the previous year (aOR: 0.72; 95% CI: 0.62–0.84; *p* < 0.001). Moreover, considering the sources of information, students consulting governmental regulation (aOR: 0.53; 95% CI: 0.40–0.71; *p* < 0.001), blogs/social networks (aOR: 0.52; 95% CI: 0.37–0.70; *p* < 0.001) or those who were not interested in searching influenza vaccination information (aOR: 0.54; 95% CI: 0.42–0.70; *p* < 0.001) were statistically associated with a lower probability of receiving influenza vaccination during the previous year. Moreover, considering themselves as a high-risk subject is the main reason associated with the highest probability of taking influenza vaccination during the previous year, whereas, avoiding spread of the infection among family members/general population or patients is associated with lower odds of receiving influenza vaccination during the previous year (aOR: 0.62; 95% CI: 0.45–0.86; *p* = 0.004 and aOR: 0.37; 95% CI: 0.26–0.53; *p* < 0.001, respectively). On the contrary, all the reasons for not taking the vaccine were statistically significantly associated with similar low odds of receiving influenza vaccination during the previous year (aORs ranged between 0.01 and 0.03). Results are shown in Table 2.

### 3.3. Influenza Vaccine Acceptance during the Following Year

For each unit increase in age, the probability of receiving the influenza vaccination during the following year increased in a statistically significantly manner (aOR: 1.06; 95% CI: 1.03–1.09; *p* < 0.001). Comparing students enrolled in a medical degree course with all the others showed that those enrolled in other health sciences programs were less likely to get vaccinated against influenza during the following year (aOR: 0.58; 95% CI: 0.46–0.73; *p* < 0.001). Moreover, considering the sources of information, students consulting governmental regulations (aOR: 0.41; 95% CI: 0.27–0.62; *p* < 0.001), blogs/social networks (aOR: 0.48; 95% CI: 0.29–0.80; *p* < 0.001) or those who were not interested in searching influenza vaccination information (aOR: 0.43; 95% CI: 0.30–0.62; *p* < 0.001) were statistically associated with a lower probability of being vaccinated during the following year.

Moreover, avoiding spread of the infection among family members/general population or patients is associated with higher odds of receiving the influenza vaccine during the following campaign (aOR: 44.56; 95% CI: 16.38–121.22; *p* < 0.001 and aOR: 16.56; 95% CI: 5.95–46.05; *p* < 0.001, respectively) when compared with considering themselves as a high-risk subject (reference). Results are shown in Table 3.

## 4. Discussion

Our study aimed at investigating the association between main reasons for accepting or refusing influenza vaccination with the decision of receiving influenza vaccination during both previous and following year. These two questions allowed us to investigate both behavior and attitude toward influenza vaccination among health sciences students gathered from various Italian universities. Over 3000 students answered our questionnaire, with 3131 correct records being collected over the course of one year.

The main finding of our study was the role of information sources and reasoning of accepting/refusing influenza vaccination in defining their attitude towards immunization. The immunization behavior for different vaccines in different groups of health sciences students has already been studied by various authors. In particular, previous studies conducted among health sciences students revealed that degree program is one of the main factors impacting knowledge, attitudes, and immunization practice. Specifically, medical students are more prone to protect themselves against influenza, more often consider themselves a high-risk group, and lastly their level of knowledge on vaccine-preventable diseases and related vaccinations was higher compared to students of other health professions [29]. Our results are similar to a 2021 Israelian study, in which medical students showed higher vaccination acceptance than nursing students, with a generally better attitude towards vaccines [29,30]. Although these findings are surely interesting, more in-depth analysis would be required in order to identify the underlying causes for both these differences and the different outcomes of all the aforementioned studies. Economic and social factors, as well as community identity and country-specific cultural phenomena may play a role in determining the behavior of students when vaccination is taken in consideration [31,32,33]. Similarly, sources consulted for up-to-date information about vaccinations are different among the various health professionals. For instance, a previous study revealed that institutional sources were consulted by two-third of pharmacists [34]. In our sample, less than 20% of the students consulted institutional webpages or scientific data (respectively) in order to make the decision on getting the influenza vaccination during the previous year. However, the percentage doubled when intention to receive influenza for the following year was considered. Information source is another important element in the decision-making process for getting vaccinated or not. Indeed, information obtained from official vaccination campaigns was associated with the highest score on vaccine literacy scale [35].

According to our findings, consideration for others and for one’s own health are the main reasons associated with higher probability of vaccination acceptance. In particular, considering themselves a high-risk subject was especially relevant to determine compliance to the previous year’s vaccination campaign, while the idea of protecting others was the most quoted among those who were willing to get vaccinated during the following season. These results are similar to those observed in a 2021 Iraqi study, which highlighted “perceived benefits” and “preventive measures [perceived utility]” as facilitators of vaccination uptake among both HCWs and the general population [36]. Indeed, the ethical importance of vaccination among HCWs has been long discussed due to the tricky relationship existing between free will and professional duty. However, the debate is currently oriented towards the need to intervene by making vaccination mandatory among HCWs [37]. Italian Public Health residents agree with compulsory vaccination, considering it a useful and effective tool aimed at increasing the vaccination coverage rate [38]. It is important to associate government regulation with iterations of the active call to non-compliant HCWs, even implementing other interventions such as declination statements and educational campaigns [39]. Moreover, propensity towards supporting mandatory vaccinations is higher among students with higher levels of vaccination knowledge [40]. From a more practical point of view, influenza vaccination is a fundamental tool to contain healthcare-related expenses, as it limits workday losses and lowers the risk of HCW-patient spread of infectious diseases [41].

From the data collected, it emerges that considering influenza as not a severe infection or considering themselves irrelevant in spreading influenza to family members and general population were the main reasons for refusing influenza vaccination (approximately 66% for past and 11% for future campaigns). On the contrary, only 6% of our sample (approximately) considered flu vaccine as not safe and effective, revealing that safety profile is not a determinant of vaccine acceptance. However, considering the high rate of under-reporting, a post-marketing surveillance program should be implemented to provide information regarding adverse events following immunization (AEFI), adverse events of special interest (AESI) and causality assessment [42].

### 4.1. Implications for Public Health Policies and Practice

The added values of our work are related to the implications for public health policies and practice. Our results offer a broad description of main factors potentially associated with acceptance/refusal of influenza vaccination among health sciences students. In particular, we assessed differences among degree course, as well as the association between information sources and main reasons for accepting/refusing influenza vaccine. Based on our results, it emerged that approximately 34% of health sciences students were not interested in searching information on influenza, and approximately 10% searched information on blogs/social networks. Moreover, searching information on blogs/social networks or those who were not interested in searching information were statistically associated with a lower probability of receiving influenza vaccination. Furthermore, considering themselves as a high-risk subject is the main reason associated with the highest probability of taking influenza vaccination during the previous year but not for the following year, where the reasons of avoiding spread of the infection among family members/general population or patients were associated with the highest probability of taking influenza vaccination.

These data are relevant for policy makers and for public health professionals involved in designing and planning vaccination campaigns. Knowing the reasons more associated with vaccine acceptance is fundamental in order to target educational messages in an effective manner. Further, health sciences’ curricula could be enriched with classes and courses about practices and attitudes towards vaccines [43], and mainstream media could be employed as a vehicle to correct information [44]. Lastly, sharing a unique and coherent message among different stakeholders using available communication tools is crucial to obtain the best results in terms of adherence to influenza vaccine [45,46]. A global educational and organizational strategy should include the phase of feedback and report, the discussion of obtained vaccination coverage and the analysis of vaccine hesitancy determinants in target populations [47].

### 4.2. Limitations and Strengths

Before generalizing our results, some criticisms should be considered. Firstly, this is a cross-sectional study and causality cannot be ascertained. Secondly, immunization status was not derived from medical records, but was self-reported. In this respect, our data might be prone to recall bias or social desirability bias. However, we used a self-administered survey that has been proved to reduce risk of social desirability bias; also limited by the anonymity of the questionnaire. Another weakness is that our dataset dates back to the pre-COVID-19 era. It is undoubted that the pandemic heavily impacted the general population’s opinion about vaccination [48]. On one hand, widespread news about illness-related mortality increased risk perception among the general population, somehow countering the “complacency” dimension of vaccination hesitancy towards both COVID-19 vaccines and others [49]. On the other hand, the pandemic-related misinformation deluge aggravated antivaccination movements and reduced trust in vaccination in general [50,51]. Keeping this in mind, it would be interesting to repeat our study in the current post-pandemic scenario in order to evaluate the effects of the COVID-19 pandemic on vaccination attitudes in health sciences students.

On the other hand, the main strength of the study is its large sample size (more than 3000 students) from 14 different universities, which allowed us to minimize the regression model’s instability and to better understand the impact of some factors on vaccination acceptance. Moreover, missing data were avoided because an online questionnaire with mandatory answers was used. Furthermore, an already available validated questionnaire was used, increasing comparability with previous studies and certainty around the measurement [52,53].

## 5. Conclusions

Health sciences students often attend hospital wards from the very beginning of their studies. Therefore, they should be considered an important target group for influenza vaccination campaigns. Focusing on the intention on getting influenza vaccination for the following year, our findings revealed that health sciences students recognize the importance of getting vaccinated to protect patients and family members, but not to protect themselves. In this respect, efforts should be made to increase their knowledge on the severity of infectious diseases for themselves, which is often underestimated, therefore leading to lower vaccination uptake. Consequently, efforts should be made to ensure that health sciences students are enabled to receive the more appropriate and targeted immunization information, preferably through official channels, as for instance their hospital or university.

## Figures and Tables

**Table 1:** Descriptive characteristics of the sample and bivariate associations. Used Pearson’s Chi-square test.

			Flu Vaccination Previous Year			Flu Vaccination Following Year		
Variables		Total n (%)	No (%)	Yes (%)	*p*-Value	No (%)	Yes (%)	*p*-Value
Gender	Female	2132 (68.09)	1908 (89.49)	224 (10.51)	0.096	1381 (64.77)	751 (35.23)	0.588
	Male	999 (31.91)	874 (87.49)	125 (12.51)		657 (65.77)	342 (34.23)	
Age	Mean ± SD	23.41 ± 3.69	23.31 ± 3.64	24.23 ± 3.94	<0.001	23.31 ± 3.63	23.59 ± 3.78	0.025
Degree Program	Medicine and Surgery	1219 (38.93)	1036 (84.99)	183 (15.01)	<0.001	737 (60.46)	482 (39.54)	<0.001
	Other	1912 61.07)	1746 (91.32)	166 (8.68)		1301 (68.04)	611 (31.96)	
Main source of information on vaccines	Institutional webpages	324 (10.35)	269 (83.02)	55 (16.98)	<0.001	181 (55.86)	143 (44.14)	<0.001
	Scientific data	810 (25.87)	670 (82.71)	140 (17.28)		449 (55.43)	361 (44.57)	
	Governmental regulation	641 (20.47)	595 (92.82)	46 (7.18)		452 (70.51)	189 (28.76)	
	Blogs/social networks	299 (9.55)	274 (91.64)	25 (8.36)		213 (71.24)	86 (28.76)	
	I do not care about	1057 (33.76)	974 (92.15)	83 (7.85)		743 (70.29)	314 (29.71)	

n: number; SD: standard deviation.

**Table 2:** Multivariable logistic regression for getting influenza vaccine during the last campaign. Adjusted Odds Ratios (aOR) are presented. Each independent variable is adjusted for gender and age. Based on 3131 observations.

		Getting Influenza Vaccine during the Previous Year		
		aOR	95% CI	*p*-Value
Gender	Female	1		
	Male	0.95	0.81–1.11	0.501
Age	As the unit increases	1.02	1.01–1.04	0.044
Degree Course	Medicine and Surgery	1		
	Other	0.72	0.62–0.84	<0.001
Main source of information on vaccine	Institutional webpages	1		
	Scientific data	1.02	0.79–1.33	0.859
	Governmental regulation	0.53	0.40–0.71	<0.001
	Blogs/social networks	0.52	0.37–0.73	<0.001
	I do not care about	0.54	0.42–0.70	<0.001
Main reason for taking the vaccine ^	I consider myself as a high-risk subject	1		
	To avoid infecting family members and general population	0.62	0.45–0.86	0.004
	To avoid infecting patients	0.37	0.26–0.53	<0.001
	Other	0.28	0.13–0.65	0.003
Main reason for not taking the vaccine *	Flu vaccine is not safe and effective	0.03	0.01–0.09	<0.001
	Flu is not a severe infection	0.01	0.01–0.02	<0.001
	I consider myself irrelevant in spreading influenza to family members and general population	0.01	0.01–0.04	<0.001
	I consider myself irrelevant in spreading influenza to patients	0.02	0.01–0.06	<0.001

aOR: Adjusted Odds Ratio; CI: Confidence Interval; ^ these data are available only for those who are intending to receive influenza vaccine; * these data are available only for those who are not intending to receive influenza vaccine.

**Table 3:** Multivariable logistic regression for getting influenza vaccine during the following campaign. Adjusted Odds Ratios (aOR) are presented. Each independent variable is adjusted for gender and age. Based on 3131 observations.

		Getting Influenza Vaccine during the Following Campaign		
Independent Variables		aOR	95% CI	*p*-Value
Gender	Female	1		
	Male	1.18	0.94–1.50	0.158
Age	As the unit increases	1.06	1.03–1.09	<0.001
Degree Course	Medicine and Surgery	1		
	Other	0.58	0.46–0.73	<0.001
Main source of information on vaccine	Institutional webpages	1		
	Scientific data	1.03	0.73–1.46	0.855
	Governmental regulation	0.41	0.27–0.62	<0.001
	Blogs/social networks	0.48	0.29–0.80	<0.001
	I do not care about	0.43	0.30–0.62	<0.001
Main reason for taking the vaccine ^	I consider myself as a high-risk subject	1		
	To avoid infecting family members and general population	44.56	16.38–121.22	<0.001
	To avoid infecting patients	16.56	5.95–46.05	<0.001
	Other	13.22	4.72–37.08	<0.001
Main reason for not taking the vaccine *	Flu vaccine is not safe and effective	0.22	0.13–0.35	<0.001
	Flu is not a severe infection	0.17	0.14–0.21	<0.001
	I consider myself irrelevant in spreading influenza to family members and general population	0.41	0.30–0.57	<0.001
	I consider myself irrelevant in spreading influenza to patients	0.45	0.29–0.68	<0.001

aOR: Adjusted Odds Ratio; CI: Confidence Interval; ^ these data are available only for those who are intending to receive influenza vaccine; * these data are available only for those who are not intending to receive influenza vaccine.

## Data Availability

Data will be made available on request.

## Supplementary Materials

The following supporting information can be downloaded at: https://www.mdpi.com/article/10.3390/vaccines11040717/s1, Table S1: Number of health sciences students according to degree programme and University center.
