# Supplementary material for: Past and Future Influenza Vaccine Uptake Motivation: A Cross-Sectional Analysis among Italian Health Sciences Students
Source: Vaccines (Basel). 2023 Mar 23;11(4):717. doi: 10.3390/vaccines11040717 (PMC10143571; doi:10.3390/vaccines11040717)
Supplement: Supplementary file 1 [file vaccines-11-00717-s001.zip › vaccines-2221136-supplementary.pdf]

Supplementary Table S1. Number of health sciences students according to degree programme and University center.

	Ancona	Bari	L'Aquila	Messina	Naples	Palermo	Parma	Pavia	Perugia	Rome	Salerno	Siena	Turin	Udine
Medicine	47	62	31	102	125	79	55	174	49	59	167	139	105	25
Healthcare Assistance	0	10	0	0	0	2	0	0	0	0	0	0	0	0
Pharmacy	0	1	1	17	21	1	0	23	95	0	0	0	0	0
Dietetics	8	11	5	0	1	0	0	1	0	4	0	0	11	0
Professional Education	0	10	0	0	0	0	0	0	0	0	0	0	9	2
Physiotherapy	0	11	0	0	11	2	7	4	37	5	3	0	39	1
Dental Hygiene	15	1	0	0	5	0	0	2	0	6	0	0	1	0
Pediatric Nursing	0	1	1	0	0	0	0	0	1	0	0	0	0	0
Nursing	35	25	9	0	52	72	30	453	90	52	13	7	71	7
Speech therapy	0	2	0	5	9	1	3	0	18	4	0	1	25	0
Dentistry	2	2	1	8	14	2	5	24	0	0	1	9	22	0
Orthoptics	2	3	1	4	4	1	0	1	0	5	0	1	7	0
Obstetrics	0	8	3	4	34	1	8	5	20	2	0	22	18	0
Podiatry	0	0	0	0	0	0	0	0	0	2	0	0	0	0
Rehabilitation Sciences for Healthcare Professionals	0	0	1	0	0	1	0	0	14	0	0	2	0	0
Healthcare Sciences Professions Prevention	9	6	1	0	9	1	4	2	20	1	0	0	1	0
Healthcare Sciences Professions Technical Assistance	0	3	0	0	3	1	0	1	0	0	1	1	3	0
Healthcare Professions Sciences Technical Diagnostic	1	4	3	3	0	1	8	4	12	4	0	0	19	1
Sport Sciences	0	8	1	0	0	3	6	11	7	9	8	1	0	1
Audioprosthetic Techniques	0	0	0	0	0	0	0	0	2	0	0	0	0	0
Cardiocirculatory Physiopathology and Cardiac Perfusion Techniques	1	1	0	0	0	0	0	2	0	2	0	0	0	0
Biomedical Laboratory Techniques	0	0	0	0	1	1	0	0	0	0	1	0	0	0

Neurophysiology Techniques	0	1	0	4	0	0	0	1	0	0	0	1	1	0
Orthotics Techniques	0	0	2	0	2	0	1	1	0	1	0	0	1	0
Radiology Techniques	0	1	0	0	7	1	1	2	3	0	10	0	0	0
Psychological Rehabilitation Techniques	0	0	0	0	0	0	0	0	1	0	0	0	0	0
Audiometric Techniques	2	4	0	0	0	0	0	0	0	5	0	1	5	0
Occupational Therapy	0	1	1	0	0	1	0	1	0	1	0	0	0	0
Neuro-Psychomotor therapy of developmental age	0	0	0	0	2	0	0	1	0	5	0	0	12	0
Environment and Workplace Prevention Techniques i	0	0	0	0	0	0	0	0	0	0	0	0	1	0
